# Onychogryphosis in tuberous sclerosis complex: an unusual feature*

**DOI:** 10.1590/abd1806-4841.20164720

**Published:** 2016

**Authors:** Xiang-chun Han, Li-qiang Zheng, Tie-gang Zheng

**Affiliations:** 1 The First Affiliated Hospital to Hebei North University – Zhangjiakou, China; 2 Chinese People's Liberation Army General Hospital – Beijing, China; 3 The 251st Hospital of Chinese PLA – Zhangjiakou, China

**Keywords:** Mild cognitive impairment, Nail diseases, Tuberous sclerosis

## Abstract

Onychogryphosis is an acquired nail plate change. It often affects the toenail and is characterized by an opaque, yellow-brownish nail plate that is distorted, grossly thickened, elongated, and partly curved resembling a ram's horn. Tuberous sclerosis complex is a multisystem disorder associated with high rates of mental retardation, autism, cognitive impairment, behavioral problems, or seizures. Nail disease can also be associated, which is a concern to patients due to pain and nail distortion. We reported a typical tuberous sclerosis complex patient with distinctive clinical features of a ram's horn nails, which presented a great challenge to surgical treatment and nail restoration.

## INTRODUCTION

Onychogryphosis is a nail deformity characterized by an opaque, yellowish-brown nail plate that is distorted, grossly thickened, elongated and partly curved like a ram's horn.^1^ Possible causes of onychogryphosis include continuous pressure and friction on the toenails due to improper footwear, trauma, ichthyosis, psoriasis, fungal infection, as well as cognitive impairment or behavioral problems.^1^ Tuberous sclerosis complex (TSC) is a multisystem disorder associated with high rates of mental retardation, autism, and seizures.^2-4^ Developmental and behavioral problems are also common in TSC. The overlap existence of onychogryphosis and TSC has not been previously documented. We reported a typical TSC patient with distinctive clinical features of a ram's horn nails, which presented a great challenge to surgical treatment and nail function restoration.

## CASE REPORT

A 66-year-old woman who had been diagnosed as having TSC at age 20. Medical history revealed hypertension (20 years duration) – treated with nifedipine sustained-release tablets (20 mg/bd/po); hyperuricemia with gout (1 year) – treated with allopurinol (100 mg mg/bd/po); and chronic renal dysfunction (6 years) without timely treatment. Up to now, epileptic seizure had been recorded twice and her daughter died of an epilepsy attack 2 years before.

**Figure 1:**
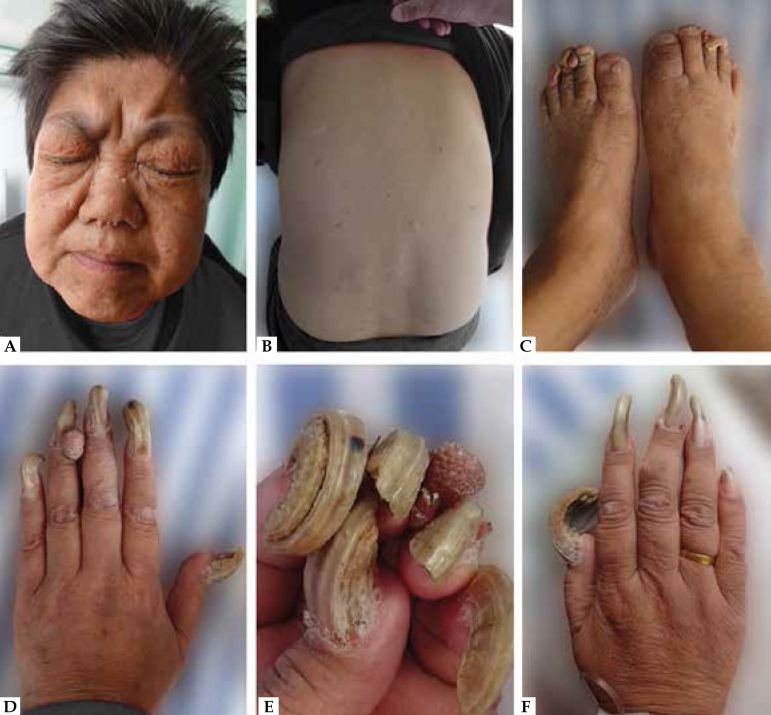
**A:** Extensive, smooth, skin-colored, papules involving the malar region in a butterfly distribution. **B:** A hypomelanotic macule and several plaques with variable size and shape located on her back. **C-F:** Multiple fusiform, strawberry-like and vermiform, periungual reddish tumors extended from the nail groove. Underlying nail plate distorted, deformed, and protracted resembling a ram’s horn

Physical examination showed extensive, smooth, skin-colored, papules involving the malar and neck regions in a butterfly distribution, measuring 2-5 mm in diameter (Figure 1). She presented with a hypomelanotic macule and several plaques with variable size and shape on her back (Figure 1). Multiple vermiform, fusiform, and strawberry-like, periungual reddish tumors extended from the nail groove; the underlying nail plate was distorted, deformed, and protracted resembling a ram's horn (Figure 1). Because of rapid growth and pain, bilateral toenail and the right second nail plates were removed by surgery 10 years before. Despite this, periungual fibroma still regrows around her left toenail.

**Figure 2:**
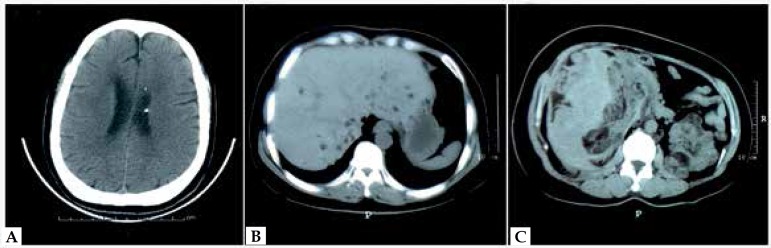
**A:** Brain CT shows two calcified nodules on the lateral ventricles. **B** and **C:** CT examination on liver and kidney revealed multiple cystic nodules suggestive of a diagnosis of myolipoma.

Histopathology of lesions from face and nail tumor revealed angiofibromas and periungual fibroma, respectively. A cranial CT scan showed several subependymal calcific nodules on the lateral ventricles (Figure 2). CT examination on liver and kidney revealed multiple cystic nodules suggestive of myolipoma (Figure 2). She showed intellectual disability (mean IQ 59) and slight cognitive impediment. Fungal cultures of skin lesions proved negative. No other clinical features for keratin mutations were found and other systemic detection is negative.

## DISCUSSION

TSC is a genetic multisystem disorder characterized by widespread hamartomas in several organs, including the brain, eyes, heart, lung, liver, kidney, and skin.^2^ Skin manifestations are the major diagnostic criteria for the diagnosis of TSC and usually a concern to patients for visiting dermatologists because of pain and distortion of the nail.^5^

Nail disorders in TSC may arise from persistent compression of ungual fibromas. In addition, individuals with TSC are at significant risk for cognitive impairment. Poor nursing care and wearing unfit shoes also contribute to nail deformation. There is evidence of a bimodal IQ distribution, with about half the TSC population having an IQ within the normal range (IQ P≥70) and most of the remainder exhibiting severe cognitive impairment (IQ < 30).^6.7^ Developmental and behavioral problems are also common in TSC.^3,7,8^

Nails are cutaneous appendages mostly involved in mechanical functions. However, the nail may reflect the presence of various systemic disorders evidenced by alteration of its shape, size, color, or texture. Genodermatoses are multisystem disorders with cutaneous involvement.^9^ Many of the genodermatoses present with nail changes and some of these may be the clinical pointers to the diagnosis. Nail lesions of TSC mainly included longitudinal nail grooves, red comets, longitudinal leukonychia, splinter hemorrhages and hypertrophic nail dystrophy.^5^

Hypertrophic nail dystrophy, the predominant clinical feature of PC, is typically noted within the first few months of life, though rarely it presents later.^10^ PC is a rare genodermatosis caused by mutations in any of the four genes KRT6A, KRT6B, KRT16, or KRT17, which can lead to dystrophic, thickened nails and focal palmoplantar keratoderma, among other manifestations.^10^ However, the character of the nail changes and the severity of the dystrophy are variable from patient to patient. A prominent thickening of the nail bed is reported, often with progressive distal elevation. The surface of the nail can be rough or smooth and some nails develop a ''pincer'' or ''omega'' pattern whereas other nails taper off prematurely before reaching the distal fingertip.^4^ The fingernails of these patients develop an apparent recession of the nail plate that leaves the distal fingertip with a slightly bulging appearance.

Other differential diagnosis mainly include onychomycosis. Onychomycosis could be a contributing factor, but this diagnosis is not adequately ruled out by either fungal culture or histological evaluation of nail clippings. Although the hyperkeratotic nail thickening seen in TSC is similar to that of onychomycosis, fungal infections do have no hereditary components.

Diagnosing TSC in our case was not difficult because of the peculiar clinical features of the disease, except for the "horn-like" hypertrophic nail plates, resembling a previous case reported by Mohrenschlager *et al.*^1^ Based on the data provided, we consider it an extremely rare feature of TSC. Poor nursing care and cognitive impairment may play a key role in nail formation. Moreover, one could imagine that subungual fibromas could have distorted or elevated the nail plate sufficiently to initiate the nail changes in our patient, but based on his preference, the lesions were not excised and pathologically evaluated, giving support to our hypothesis.
